# Autophagy: machinery and regulation

**DOI:** 10.15698/mic2016.12.546

**Published:** 2016-12-01

**Authors:** Zhangyuan Yin, Clarence Pascual, Daniel J. Klionsky

**Affiliations:** 1 Life Sciences Institute, and Department of Molecular, Cellular and Developmental Biology; University of Michigan, Ann Arbor, MI, USA 48109.

**Keywords:** autophagy, autophagosome formation, physiological roles, cellular homeostasis, pathogenesis, physiological roles, regulation

## Abstract

Macroautophagy/autophagy is an evolutionarily conserved cellular degradation process that targets cytoplasmic materials including cytosol, macromolecules and unwanted organelles. The discovery and analysis of autophagy-related (Atg) proteins have unveiled much of the machinery of autophagosome formation. Although initially autophagy was regarded as a survival response to stress, recent studies have revealed its significance in cellular and organismal homeostasis, development and immunity. Autophagic dysfunction and dysregulation are implicated in various diseases. In this review, we briefly summarize the physiological roles, molecular mechanism, regulatory network, and pathophysiological roles of autophagy.

## INTRODUCTION

Cellular homeostasis requires a proper balance between synthesis and degradation. The two major degradative pathways for cellular components in eukaryotic organisms are autophagy and the proteasome. Autophagy (“self-eating”) is the bulk degradation of long-lived cytosolic proteins and organelles; whereas the ubiquitin-proteasome degradative system is primarily responsible for the turn-over of short-lived proteins. There are distinct types of autophagy, which vary from each other based on the inducing signals and temporal aspects of induction, type of cargo and mechanism of sequestration. One of the fundamental differences between different types of autophagy is that they can be selective or nonselective. Macroautophagy (hereafter autophagy) is the best characterized pathway and involves the formation of a transient double-membrane structure, the phagophore, that is the active sequestering compartment. Following expansion and closure this structure becomes an autophagosome that subsequently fuses with the lysosome/vacuole leading to the degradation of the cargo 1,2.

In this review, we discuss the physiological roles of autophagy including its function in protein and organelle quality control, development, cell death and immunity. We also describe the morphology and machinery underlying the formation of the autophagosome, which can be summarized in five distinct events: induction, nucleation, expansion, fusion and cargo degradation/recycling. Next, we distinguish among the different mechanisms used to regulate autophagy, and conclude with a brief discussion concerning pathological connections with this process.

## PHYSIOLOGICAL ROLES OF AUTOPHAGY

As a highly conserved survival mechanism of all eukaryotic cells, autophagy primarily acts as an adaptive response to environmental adversity, especially starvation, one of the most common threats to many organisms. When there is no food available, or when resources become limited, cells will start to degrade and recycle macromolecules including proteins, lipids and carbohydrates for the synthesis of essential components and as an energy supply. One of the main mechanisms available for this purpose is autophagy. With the discovery of the autophagy-related (*ATG*) genes in yeast and subsequent in-depth studies in various animal and cellular models, many additional physiological processes have been linked to autophagy including intracellular quality control, maintenance of cellular and tissue homeostasis, anti-aging, cell differentiation and development, and innate and adaptive immunity.

### Protein/organelle quality control and cellular homeostasis

Autophagy induced by nutrient deprivation or metabolic perturbations is relatively nonselective, and essentially any part of the cytoplasm can be recycled via this bulk degradative pathway. Conversely, autophagy can be highly selective to facilitate disposal of damaged or surplus structures before they become toxic to the cells 3. This latter type of autophagy is characterized by the presence of degradation cues, typically including a ligand on the target, and the involvement of selective autophagy receptors along with at least one scaffold protein 4,5. Selective autophagy targets cargoes including protein aggregates, mitochondria, peroxisomes, endoplasmic reticulum, bacterial pathogens and signaling complexes when they are no longer needed 6. One of the best-characterized selective autophagy-like mechanisms is the yeast cytoplasm-to-vacuole targeting (Cvt) pathway, which utilizes the core machinery of autophagy to deliver the precursor form of the hydrolase aminopeptidase I, along with other degradative enzymes, to the vacuole 7.

Although much attention has been paid to induced autophagy that occurs under different stress conditions, constitutive turnover of cytoplasmic contents by basal autophagy, even during favorable growth conditions, is also crucial for proper cell physiology. This low level of autophagy works in part as a quality-control mechanism, and is especially vital for homeostasis of post-mitotic cells such as hepatocytes and neurons. Genetic studies have revealed that failures in basal autophagy are associated with neurodegenerative disease, cancer and inflammation. For example, mice deficient for ATG7 in pancreatic epithelial cells develop severe pancreatic inflammation and extensive fibrosis 8, neural cell-specific *Atg5* knockout mice show accumulation of abnormal proteins in neurons and exhibit deficits in motor function 9, and the decreased expression of mitophagy genes leads to unwarranted inflammation 5. Therefore, autophagy works as a cellular housekeeper in normal physiological conditions. Accumulation of misfolded and oxidatively damaged proteins, as well as dysfunctional organelles such as mitochondria, is not only a sign of but also one of the causes of aging 10. Accordingly, the clearance of protein aggregates and improperly functioning organelles helps improve cellular function, extend lifespan and avoid cell death. Indeed, autophagy is suggested to confer anti-aging effects. On the one hand, defective autophagy is associated with degeneration and premature aging. On the other hand, increased autophagy at the whole-body level contributes to longevity in different model organisms 11. Although the specific mechanism through which autophagy might contribute to anti-aging remains unknown, the modulation of this pathway is still considered to be a promising target for improving healthy aging.

### Development

The capability of autophagy to respond to external cues rapidly, and to modify intracellular architecture enables it to be the crucial mechanism for cellular remodeling during animal development 12. The generation of autophagy-defective mutants in various model organisms has shown the important roles autophagy plays during development. For example, autophagy participates in sporulation in yeast, it is necessary for dauer formation and the degradation of P granules in *C. elegans* somatic cells, and in *Drosophila* certain autophagy mutants show larval lethality or failure in metamorphosis 13. In mammals, autophagy is vital for pre-implantation embryo development, the survival of neonates and organogenesis. After fertilization, autophagy along with the ubiquitin-proteasome system disposes of sperm mitochondria, and thus contributes to heteroplasmy 14. After the late two-cell stage, autophagy is highly activated, targeting maternal mRNA and proteins, which might be necessary for zygotic genome activation 15. *Atg5* knockout mice with the elimination of maternally derived ATG5 produce embryos that never go beyond the eight-cell stage.

Another example of the role of autophagy at an organismal level can be seen in newborn mice. Following birth, the placental nutrient supply from the mother suddenly terminates, which challenges the neonates with severe starvation; autophagy appears to play a critical role during the transition to breast feeding by supplying nutrients, although it is also possible that neurological defects associated with the absence of autophagy result in an inability to breast feed 16. During late stages of embryonic and postnatal development, autophagy also plays an important role in cardiogenesis, osteogenesis, central nervous system development and cell differentiation 13. A representative example of the role of autophagy is seen with erythropoiesis. Mature erythrocytes are generated from erythroblasts and are devoid of most cellular organelles. The elimination of mitochondria, ribosomes and other organelles that are no longer necessary for cellular function is partly dependent on autophagy regulated by multiple modulators 17.

### Crosstalk with cell death

During the development of *Drosophila*, autophagy is found to facilitate cell death while removing obsolete tissues, suggesting a dual role for this primarily cytoprotective process in physiological conditions. Accordingly, there is extensive crosstalk between autophagy and apoptosis with regard to cell fate determination. Both processes are downstream of common signals, such as those involving TP53/p53 and BH3-only proteins, and they share common regulatory components including BCL2, as well as mutually regulating each other 18. Autophagy reduces the possibility of apoptosis by carrying out mitophagy, and through the specific targeting and degradation of pro-apoptotic proteins 19. In turn, the activation of apoptosis inhibits autophagy, as caspases can cleave and inactivate essential autophagy proteins. In some cases, the cleavage of autophagic proteins even converts them into pro-apoptotic proteins 19. In a simple model, autophagy precedes apoptosis as a first response to cellular damage; if unsuccessful in eliminating the damage, autophagy is blocked and apoptosis is induced.

The relationship between these two processes can be highly context dependent. For example, during *Drosophila* larval metamorphosis, the removal of the salivary gland is dependent on both autophagy-mediated cell death and apoptosis, whereas degradation of the mid-gut only relies on autophagy 20. During ovary development, caspases are needed for autophagy induction under stress conditions, whereas nurse cell apoptosis requires autophagic clearance of its inhibitor, Bruce 21.

It is not difficult to imagine that autophagy can participate in cell death, especially when the process is purposely dysregulated for therapeutic purposes. However, it is important to note that in most organisms autophagy is primarily a protective response. To demonstrate that autophagy is causative of cell death (and in particular has not been induced during stress to prevent cell death), autophagic cell death, formerly termed type II cell death, it is imperative to show that the cell death was caused by and was dependent on autophagy, meaning that it will be suppressed by the chemical or genetic inhibition of autophagy activity 22. Examples of autophagic cell death include autosis, which is regulated by the Na^+^, K^+^-ATPase pump and can be induced by starvation and autophagy-inducing peptides 23, and ferroptosis, in which autophagy degrades the cellular iron storage protein ferritin and leads to the accumulation of reactive oxygen species inside the cell and consequently cell death 24.

### Immunity

Considering the function of autophagy in organelle elimination, it is not surprising it has evolved as a primary form of innate immunity against microbial invasion. This cell-autonomous defense mechanism, termed xenophagy, is able to selectively recognize intracellular microbes including bacteria, viruses, and protozoa, and target them to the autophagic machinery for degradation 25. In fact, autophagy plays various roles in immunity including control of the pro-inflammatory response, assisting adaptive immunity through the regulation of immune system development and homeostasis, and in antigen presentation 26. The involvement of autophagy in multiple diseases will be further discussed in this review.

## MORPHOLOGY AND MACHINERY

**Figure 1:**
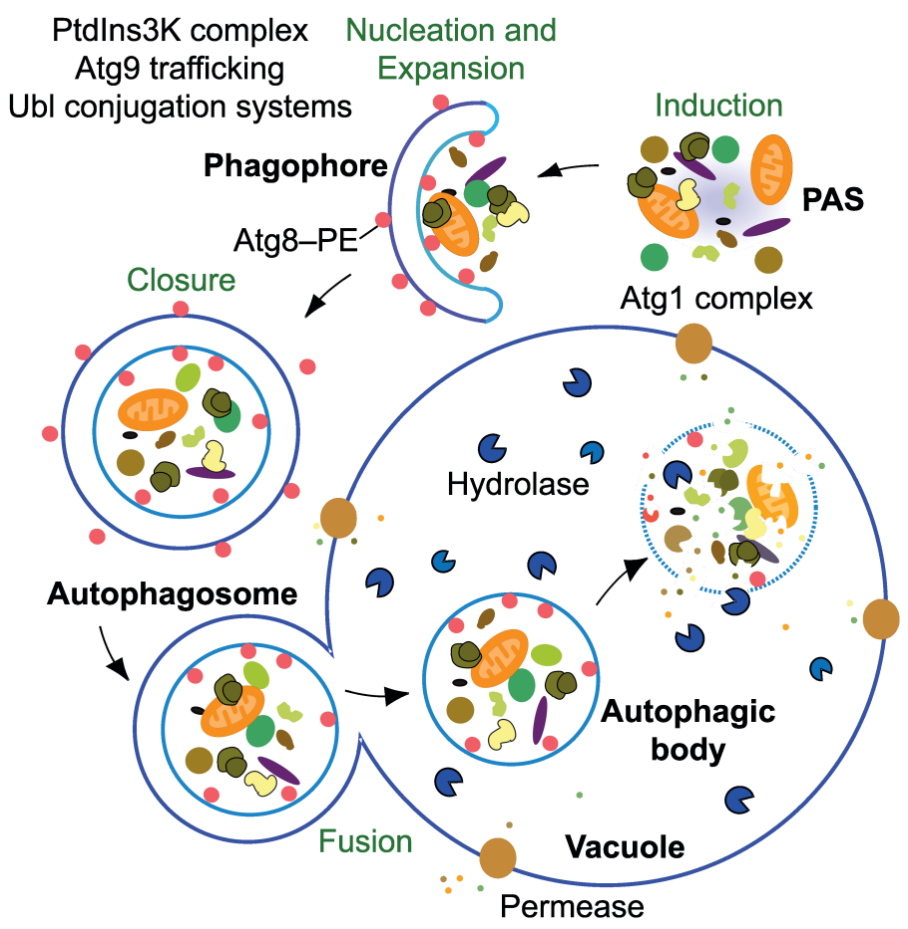
Figure 1: the mechanistic features of yeast autophagy. Initiation of autophagy requires the formation of the Atg1 kinase complex at the PAS to allow the recruitment and activation of other Atg proteins. In yeast there is typically one PAS per cell, but the precise nature of this site is not well defined; the PAS may literally become a phagophore, making it a dynamic structure that continuously reforms. Next, the PtdIns3K complex translocates to the PAS to begin the nucleation process that will catalyze further movement of additional Atg proteins to this site. Ubl proteins such as Atg8–PE participate in cargo recognition during selective types of autophagy, and also play a role in determining the size of the autophagosome. Membrane delivery to the phagophore allows expansion and maturation into an autophagosome. This process also employs the transmembrane protein Atg9; however, the mechanism for phagophore expansion is poorly understood. Upon autophagosome completion, Atg4 deconjugates Atg8–PE on the surface of the autophagosome and the resulting vesicle fuses with the vacuole. In yeast, the inner vesicle is released into the lumen as an autophagic body. Within the vacuole, the contents of the autophagic body are released following lysis by the putative lipase Atg15. Finally, the cargo is degraded by resident hydrolases, and the resulting macromolecules are then released back into the cytosol via permeases.

One of the most exciting topics in the field of autophagy today is understanding the molecular details of autophagosome biogenesis. In many ways, the sequestration step is the most complex part of autophagy; cytoplasm must be segregated, often in a directed or specific manner, and moved from the intracellular space into the vacuole/lysosome lumen, which corresponds to the extracellular space. Thus, sequestration involves an essentially double-membrane intermediate, the phagophore (Figure 1); the use of a double-membrane structure in effect, changes the topology of the cargo because subsequent fusion releases it into the lumen of the degradative compartment. The formation of the phagophore and its subsequent maturation to become the autophagosome is a transient event, but extremely dynamic, involving multiple protein complexes, that participate in the different stages of autophagy, and the mobilization of substantial membrane reserves. The stages of autophagosome biogenesis can be summarized into five events: induction, nucleation, expansion, fusion and cargo degradation/recycling. In this section of the review, we provide the details for each of these steps and briefly discuss the machinery involved.

### Induction and nucleation

#### Atg1 kinase complex and induction

Autophagy may be induced as a response to a change in the extracellular environment of a cell, and the target of rapamycin complex 1 (TORC1) is one of the signaling pathways that plays a primary role in sensing the shift in nutrient availability. Nutrient starvation, in particular nitrogen and/or amino acid limitation, initiates an intracellular signaling cascade by discontinuing TORC1 stimulation, resulting in the activation of the Atg1 kinase complex. The Atg1 kinase complex works directly downstream of the TORC1 pathway and it consists of Atg1, the regulatory protein Atg13, and a scaffold subcomplex that includes Atg17-Atg31-Atg29 (Figure 1) 27. Assembly of this complex is crucial for autophagy because it plays a role in recruiting other Atg proteins to the phagophore assembly site (PAS) and activating downstream targets through phosphorylation 28,29. Protein kinase A (PKA) is another negative regulator of the Atg1 kinase complex, in this case primarily in response to carbon source, whereas the energy sensor Snf1/AMP-activated protein kinase (AMPK) acts as a positive regulator.

#### Class III phosphotidylinositol 3-kinase (PtdIns3K) complex and nucleation

In autophagy, nucleation refers to the process of mobilizing a small group of molecules to the PAS; the phagophore is the active sequestering compartment of autophagy. In part, the nucleation process may be viewed as an amplification event that results in the further recruitment of proteins that are needed for phagophore expansion. The class III PtdIns3K complex I, which is employed specifically for autophagy, is one of the key complexes that are recruited to the PAS upon induction of autophagy. This complex is comprised of five distinct proteins: the lipid kinase Vps34, the regulatory kinase Vps15, Vps30/Atg6, Atg14 and Atg38, which are all necessary for autophagy (Figure 1) 30,31. In brief, the class III PtdIns3K is responsible for the production of phosphatidylinositol-3-phosphate (PtdIns3P) directly from phosphatidylinositol 32. This PtdIns3P is important for the correct localization of some of the Atg proteins including Atg18 and Atg2, which enables the recruitment of Atg8, Atg9 and Atg12 to the PAS 33.

### Phagophore expansion

#### Ubiquitin-like (Ubl) conjugation systems and expansion

A characteristic feature of autophagy is the formation of double-membraned vesicles known as autophagosomes, which correspond to the mature form of the phagophore (Figure 1) 34. It is important to note that phagophores are relatively transient, even relative to autophagosomes. As a result, much attention has focused on the latter, even though the autophagosome is essentially a terminal compartment that does little more than fuse with the vacuole; in other words, formation of, and sequestration by, the phagophore are the truly dynamic steps of autophagy.

There are two essential ubiquitin-like (Ubl) conjugation systems that are necessary for phagophore expansion and these involve the Ubl proteins Atg12 and Atg8 35; these two proteins have structural similarity to ubiquitin, but are not actual homologs. Atg12 is conjugated to Atg5 via the action of the E1 and E2 enzymes Atg7 and Atg10, and this conjugate binds Atg16 to form the dimeric Atg12-Atg5-Atg16 complex; there is no known E3 enzyme required for Atg12 conjugation to Atg5. Atg8 undergoes a different type of conjugation, being covalently attached to the lipid phosphatidylethanolamine. The generation of Atg8-PE involves the protease Atg4, Atg7 as an E1 enzyme and Atg3 as an E2 enzyme, with the Atg12-Atg5-Atg16 complex participating as an E3 enzyme 35, although the latter is not absolutely required for conjugation to occur 36. A detailed mechanism in which these conjugation systems operate along with other complexes to enlarge the phagophore is currently an on-going research topic. Studies have linked Atg2 and Atg18 to the proper recruitment of both Ubl proteins to the PAS, but whether Atg2 and/or Atg18 directly recruit Atg8 and Atg12 is not yet known 37.

#### Atg9 trafficking

Unlike other vesicles in the cells, the autophagosome is often referred to as being made *de novo*. This term is used to distinguish autophagosome formation from vesicle budding, which occurs throughout the secretory pathway and during endocytosis. One useful way to think about the distinction between autophagy and other vesicle-mediated processes is that vesicles used in the secretory pathway are generally of a uniform size and bud off from a pre-existing organelle already containing their cargo. The phagophore may be generated by vesicular addition (although this is a controversial topic), but it may be variable in size, in part depending on the cargo. A common view in the autophagy field is that Atg9 functions in some manner as the membrane transporter for the growing phagophore, but direct evidence or a mechanistic explanation are not available. Nonetheless, Atg9 is a good candidate for this role for multiple reasons. First, Atg9 is the only transmembrane protein that is essential for phagophore expansion 38. Second, Atg9 is found to be highly mobile in the cytosol upon rapamycin treatment 39. Third, this protein is capable of binding with itself and appears to transit to the PAS as part of a complex 40. While none of these studies directly proves the role of Atg9 in membrane shuttling, researchers have begun identifying the machinery that is involved in Atg9 trafficking. These components include Atg11, Atg23 and Atg27, which transit along with Atg9 from putative membrane donor sites to the PAS.

### Autophagosome targeting, docking and fusion

Upon completion of the autophagosome, it targets to, tethers/docks and then fuses with the vacuole. This fusion allows the release of the inner autophagosome vesicle into the vacuole lumen where it is now termed an autophagic body. Note that mammalian cell lysosomes are generally smaller than autophagosomes so autophagic bodies are not a general feature of autophagy in most of the more complex eukaryotes. The mechanism that controls the timing of fusion is not known at present; however, there are regulatory mechanisms in place to prevent premature autophagosome fusion with the vacuole, which would prevent delivery of the cargo into the vacuole lumen. For example, Atg8-PE undergoes a secondary cleavage event by Atg4, a cysteine protease that is also required for the early stages of Atg8 conjugation 41. This cleavage event (termed deconjugation) is necessary for the autophagosome to initiate its fusion with the vacuole 42. One suggestion is that deconjugation is the trigger for disassembly of the Atg proteins from the completed autophagosome, a step that presumably precedes fusion. Other cellular processes that also deliver their cargo to the vacuole employ similar components that facilitate fusion including SNARE proteins and those involved in the homotypic fusion and vacuole protein sorting (HOPS) pathway 43.

### Cargo degradation and recycling

After the cargo is delivered inside the vacuole, the autophagic body membrane is degraded by a putative lipase, Atg15 (Figure 1) 44,45, followed by cargo degradation by resident hydrolases. Once degraded, the resulting macromolecules are released back into the cytosol through various permeases including Atg22 (Figure 1) 46.

## REGULATION OF AUTOPHAGY 

Given the important roles autophagy plays in the maintenance of cellular homeostasis and survival under stress conditions and its involvement in various aspects of animal development and pathophysiology, it is not surprising that autophagy needs to be finely regulated to avoid either excessive or insufficient activity. Numerous studies have been focusing on how autophagy is kept at basal levels in normal conditions and how it is quickly switched on upon certain types of stimulation. Less well understood are the mechanisms that downregulate and prevent excessive autophagy when cells are maintained under stress conditions. Similar to the mechanism of autophagy itself, the regulatory network shows a lot in common across a broad spectrum of organisms from yeast to mammals, although the inducing signals can be more complicated in more complex eukaryotes. Nonetheless, studies in yeast have pioneered our understanding of this regulatory network.

### Nitrogen-dependent regulation

It was known that glucose or amino acid deprivation will induce autophagy long before the identification of the *ATG *genes 47,48. The primary sensor of amino acid and nitrogen change is TOR or the mammalian homolog MTOR (mechanistic target of rapamycin [serine/threonine kinase]), which is the main negative regulator of autophagy. TOR/MTOR is a conserved serine/threonine kinase that senses and integrates multiple environmental signals to inhibit catabolism and coordinate cell growth. MTORC1 can be activated by cues including energy status, nutrient levels, growth factors and amino acids 49. For yeast in nutrient-rich conditions, TORC1 directly phosphorylates Atg13, Atg1 and Atg14, which prevents the formation and/or activation of the Atg1-Atg13-Atg17-Atg31-Atg29 complex and suppresses the autophagy-specific PtdIns3K, thus inhibiting autophagy induction 50,51. In mammalian cells, amino acids are sensed by the vacuolar-type H^+^-translocating ATPase, which is present in the lysosome membrane, in conjunction with RRAG proteins and the Ragulator complex 52, which can coordinately direct MTORC1 to the lysosome membrane where it becomes activated by the GTPase RHEB 53. Under conditions of amino acid withdrawal, or treatment with the inhibitors rapamycin or Torin1, MTORC1 is suppressed and autophagy becomes derepressed. In addition to MTORC1 inactivation, nitrogen starvation results in the dephosphorylation of ULK1 (a homolog of yeast Atg1) through protein phosphatase 2A 54.

In addition to these types of post-translational regulation, transcriptional regulators also function in response to nitrogen and amino acid depletion. For example, yeast Gcn2 is a kinase that is able to sense the level of amino acids; once activated, Gcn2 induces the transcription of specific *ATG* genes including *ATG1* through the activation of the transcription factor Gcn4 55. In mammals, inactivation of MTORC1 allows the unphosphorylated form of TFEB (transcription factor EB) to translocate to the nucleus, which similarly results in the transcription of various genes involved in autophagic degradation 56.

### Energy/glucose-dependent regulation

Regulation of autophagy by glucose metabolism and energy level is also vital for cellular homeostasis. In the presence of glucose, PKA is activated by binding with cAMP. PKA then phosphorylates Atg1 and Atg13, which prevents the localization of Atg13 to the PAS 57. In addition, PKA can inhibit autophagy by direct phosphorylation of TORC1 or in mammalian cells by indirect activation of MTORC1 through inhibition of AMPK. AMPK is the major energy sensor in the cell and it is activated by an increased AMP:ATP ratio, which is one outcome of glucose depletion or other types of stress such as mitochondrial dysfunction 58. When AMPK senses low energy, it promotes autophagy by inhibiting MTOR activity through direct negative phosphorylation of RPTOR/raptor, a subunit of the MTORC1 complex, or phosphorylation and activation of the TSC1/2 complex, a negative regulator of MTORC1 59. Moreover, AMPK is able to activate ULK1 by direct phosphorylation 60. Intriguingly, this activation can be suppressed by MTORC1-dependent phosphorylation of ULK1 and a negative feedback phosphorylation of AMPK by ULK1. Similar to nitrogen deprivation, glucose starvation can activate the transcription of certain *ATG *genes by deacetylation of transcription factors including FOXO1 and FOXO3 61.

### Other types of regulation

Some types of lipid metabolism are closely related to autophagy. Free fatty acids stimulate autophagy through EIF2AK2/PKR-dependent activation of EIF2S1/eIF2α, MAPK8 (mitogen-activated protein kinase 8) or inhibition of MTORC1. Dietary lipid existing in the form of intracellular droplets will induce autophagy and be captured and delivered to lysosomes for degradation. The breakdown product, in particular fatty acids, will then mediate autophagy to avoid potential lipotoxicity 62.

Additional stimuli that are capable of inducing autophagy include hypoxia, ER stress, the amino acid metabolite ammonium, depletion of iron and the absence of growth factors. These environmental cues and relevant pathways can be more interconnected and complex at the tissue or body level. To further explore the downstream targets of the master regulators such as MTORC1 and how each ATG protein is specifically regulated, researchers have started to screen and analyze the transcription factors involved in autophagy regulation. Several transcription factors have been identified and characterized. For example, in yeast Ume6 negatively regulates *ATG8* and the inhibitory phosphorylation of Ume6 is partly responsible for the dramatic increase of *ATG8* transcripts upon starvation, which in turn controls the size of autophagosomes 63. Pho23 controls *ATG9 *transcript levels, thereby regulating the number of autophagosomes 64.

## PATHOLOGY

As discussed above, autophagy plays critical roles in maintaining normal cellular physiology. Accordingly, it is not surprising that defects in autophagy have been linked to a wide range of diseases 65,66. In fact, autophagy plays such a fundamental role in cellular health that defects in this process have been linked a vast array of pathophysiologies, which are beyond the scope of this review. Here, we briefly highlight one example of the connection between autophagy and neurodegeneration, which illustrates the complexities of manipulating this process for disease treatment.

Autophagic dysfunction has been associated with a large number of neurodegenerative diseases. One of the hallmarks of many neurodegenerative diseases is the accumulation of aggregated proteins that escape the degradative process resulting in neuronal cell death 67,68. One of the basic concepts is that fully differentiated nondividing cells rely extensively on autophagy to remove waste products that accumulate over time. Thus, neurons are particularly dependent on autophagic degradation. Most neurodegenerative diseases are age related, making it difficult to demonstrate a direct correlation between defective autophagy and the disease phenotype; however, genetically impaired autophagic flux in the central nervous system of otherwise healthy mice results in symptoms of neurodegeneration, suggesting a direct link between the two 9,69.

Alzheimer disease is one example of neurodegeneration associated with autophagy. Amyloid plaques composed of amyloid-β peptides and hyperphosphorylated MAPT/tau proteins are the most commonly known marker of the disease 70; although, as with most neurodegenerative diseases, the actual cause of toxicity is not known. Nonetheless, a general model is that defects in autophagy, which generally declines with age, may lead to an increase in these neuropeptides, ultimately resulting in neuronal cell death. Along these lines, autophagosomes have been seen to accumulate in samples from patients with Alzheimer disease, which suggests a block in the degradative system 71. Conversely, it has been proposed that in diseased cells the mammalian autophagosome may actually contribute to the generation of disease-causing peptides 72, implicating autophagy as playing a more active role in disease onset. Thus, this observation provides one example of the basic conundrum of attempting to manipulate autophagy. That is, autophagy is essential, yet too much autophagy can be lethal. Similarly, the induction of autophagy in aged organisms may have beneficial consequences, but there is also the possibility that it can promote certain disease conditions. Thus, until we know much more about this process, we must be extremely cautious in the use of autophagy-modulating drugs in disease treatment.

## CONCLUDING REMARKS

Autophagy, a tightly regulated intracellular degradation process, is crucial for cellular homeostasis, survival and organismal development, and autophagic dysfunction is linked to numerous diseases. Despite the tremendous progress made in the last two decades, our understanding of autophagy is still relatively primitive and there are many questions remaining to be answered.

During autophagy, various autophagy-related proteins undergo post-translational modification. However, how this regulation affects the overall activity of selective autophagy remains unclear. In recent years, researchers have discovered several forms of noncanonical autophagy, which only needs a subset of ATG proteins and bypasses some of the otherwise “essential” complexes. In this regard, it will be intriguing to know what the relevance is among these different mechanisms and, along a related line, how the ATG proteins gained pleiotropic functions. Some detailed aspects of autophagy also need further investigation. For example, what are the sources of the phagophore membrane? How is membrane incorporated into the expanding phagophore? Is there any type of coordinate regulation among donor membrane sources? Additional studies on the structure and interactions of the autophagy-related proteins might shed light on the above questions.

Another key question is how autophagy machinery exhibits cell- or tissue-type specificity. Each cell type has its own structural and functional characteristics and they also may require autophagy in different developmental contexts. For example, red blood cells and neurons both rely on autophagy, but for different purposes and at different times of development. How is autophagy differentially regulated in different cell types to allow proper cell differentiation and to maintain homeostasis of cells experiencing variable environments? How is the crosstalk among different regulatory pathways initiated by distinct stimuli and how is regulation coordinated? In addition, when studying the regulatory network in transformed cell lines, there is a concern that the artificial stress conditions might fail to adequately mimic the true physiological conditions. Therefore, establishing a method to monitor autophagy activity *in vivo* in a real-time manner and generating specific chemicals to inhibit or induce autophagy without apparent deleterious side effects will be particularly significant. Undoubtedly, an improved understanding of autophagy will provide more therapeutic targets for pharmaceutical or genetic strategies against disease.
